# Supplementary material for: Infection with endosymbiotic Spiroplasma disrupts tsetse (Glossina fuscipes fuscipes) metabolic and reproductive homeostasis
Source: PLoS Pathog. 2021 Sep 16;17(9):e1009539. doi: 10.1371/journal.ppat.1009539 (PMC8478229; doi:10.1371/journal.ppat.1009539)
Supplement: S1 Appendix — (DOCX) [file ppat.1009539.s001.docx]

**S1 Table.** Mapping of reads on the reference genome.

| Sex | *Spiroplasma* status | Sample ID | # reads mapped | # reads in total | % reads mapped |
| --- | --- | --- | --- | --- | --- |
| Male | - | *Spi*- male 1 | 46958358 | 73719478 | 63.698712 |
|  |  | *Spi*- male 3 | 45550127 | 91676858 | 49.6855237 |
|  | + | *Spi*+ male 2 | 50870264 | 90252960 | 56.3640949 |
|  |  | *Spi*+ male 4 | 56167457 | 82920760 | 67.7363027 |
|  |  | *Spi*+ male 5 | 55569101 | 83561518 | 66.5008276 |
| Female | - | *Spi*- female 1 | 70439889 | 95515532 | 73.7470519 |
|  |  | *Spi*- female 2 | 54270727 | 74207510 | 73.1337394 |
|  |  | *Spi*- female 3 | 60999714 | 76871714 | 79.3526134 |
|  | + | *Spi*+ female 4 | 157445206 | 226057488 | 69.6483038 |
|  |  | *Spi*+ female 5 | 55274809 | 68301764 | 80.927352 |

**S2 Table.** Number of genes that are differentially expressed between *Spi*+ and *Spi*- males in the sex-biased gene categories. Fisher’s exact tests were conducted to evaluate the significance in each sex-biased gene category.

|  | | Male | | | | | |
| --- | --- | --- | --- | --- | --- | --- | --- |
|  |  | *Spi*+ up-regulated | | | *Spi*+ down-regulated | | |
| Sex bias | # genes expressed | # genes | Odd ratio | Confidence interval (95%) | # genes | Odd ratio | Confidence interval (95%) |
| Female-biased | 2288 | 31 | 1.30 | 0.83 - 1.99 | 13 | 0.73 | 0.37 - 1.35 |
| Male-biased | 2164 | 41 | 2.08 | 1.39 - 3.10 | 41 | 4.41 | 2.74 - 7.12 |
| Unbiased | 6088 | 45 | 0.45 | 0.31 - 0.67 | 23 | 0.31 | 0.18 - 0.52 |
| Total | 10540 | 117 |  |  | 77 |  |  |

**S3 Table.** Number of genes that are differentially expressed between *Spi*+ and *Spi*- females in the sex-biased gene categories. Fisher’s exact tests were conducted to evaluate the significance in each sex-biased gene category.

|  | | Female | | | | | |
| --- | --- | --- | --- | --- | --- | --- | --- |
|  |  | *Spi*+ up-regulated | | | *Spi*+ down-regulated | | |
| Sex bias | # genes expressed | # genes | Odd ratio | Confidence interval (95%) | # genes | Odd ratio | Confidence interval (95%) |
| Female-biased | 2288 | 48 | 13.31 | 7.08 - 26.82 | 1 | 0.02 | 0.0004 - 0.09 |
| Male-biased | 2164 | 2 | 0.13 | 0.02 - 0.50 | 234 | 226.01 | 87.08 - 838.95 |
| Unbiased | 6088 | 11 | 0.16 | 0.08 - 0.31 | 3 | 0.01 | 0.002 - 0.03 |
| Total | 10540 | 61 |  |  | 238 |  |  |

|  | **S4 Table.** PCR primers used in this study. |  |  |
| --- | --- | --- | --- |
|  | gene | primer sets | Tm (°C) |
|  | *sperm-specific dynein intermediate chain (sdic)* | F, 5'-CGTGAGGAGAAGGAGAGACG-3' | 56 |
|  |  | R, 5'-TTGACCAGCGGTCATCATAA-3' | 56 |
|  | *Spiroplasma 16s rRNA-1* | F, 5’-GGGTGAGTAACACGTATCT-3’ | 55 |
|  |  | R, 5’- CCTTCCTCTAGCTTACACTA-3’ | 56 |
|  | *Spiroplasma 16s rRNA-2* | F, 5’-GCCTAATACATGCAAGTCGAAC-3’ | 59 |
|  |  | R, 5’- TAGCCGTGGCTTTCTGGTAA- 3’ | 59 |
|  | *Spiroplasma rpoB* | F, 5’-CGGTATCAGCGAAGTGTTCA-3’ | 58 |
|  |  | R, 5’- GTTCGTCGTGGCTCTCTAAAT-3’ | 58 |
|  | *Wigglesworthia recA* | F, 5'-TGCGTTTGGGTGAAGATAGAT-3' | 58 |
|  |  | R, 5'-CGTCCCATCGGTAAACCTCC-3' | 58 |
|  | *Sodalis rplB* | F', 5'-ATCGTGGCTCGTGATGGC-3' | 57 |
|  |  | R, 5'-GAACGGTCGGACGGATGC-3' | 57 |
|  | *Gff b-tubulin* | F, 5’-ACGTATTCATTTCCCTTTGG-3’ | 55 |
|  |  | R, 5’-AATGGCTGTGGTGTTGGACAAC-3’ | 55 |
|  | *Gff gapdh* | F, 5'-CTGATTTCGTTGGTGATACT-3' | 58 |
|  |  | F, 5'-CCAAATTCGTTGTCGTACCA-3' | 58 |
|  | *GpCAG* | F, 5’-ATTTTTGCGTCAACGTGA-3’ | 59 |
|  |  | R, 5’-ATGAGGATGTTGTCCAGTTT-3’ | 59 |
|  | *pgrp-la* | F, 5’-ACGACTACGAGCACGACAG-3’ | 58 |
|  |  | R, 5’-GCCGACAACCACAATCACTAAT-3’ | 58 |
|  | *male-specific protein* (GFUI008563-RA^1^) | F, 5'-TTTGGCCGCCACTGGGATTT-3' | 55 |
|  |  | R, 5'-GGGGCCTCACAGGTATTGGC-3' | 55 |
|  | *milk gland protein 1* (GFUI006902-RA^1^) | F, 5'-TGTTGGGTCGTCTTAGTGCT-3' | 56 |
|  |  | R, 5'-TGGTGGGGAAGTGATGTTCC-3' | 56 |
|  | ^1^Vectorbase gene ID. |  |  |
