# Supplementary figures and images for: Infection with endosymbiotic Spiroplasma disrupts tsetse (Glossina fuscipes fuscipes) metabolic and reproductive homeostasis
Source: PLoS Pathog. 2021 Sep 16;17(9):e1009539. doi: 10.1371/journal.ppat.1009539 (PMC8478229; doi:10.1371/journal.ppat.1009539)

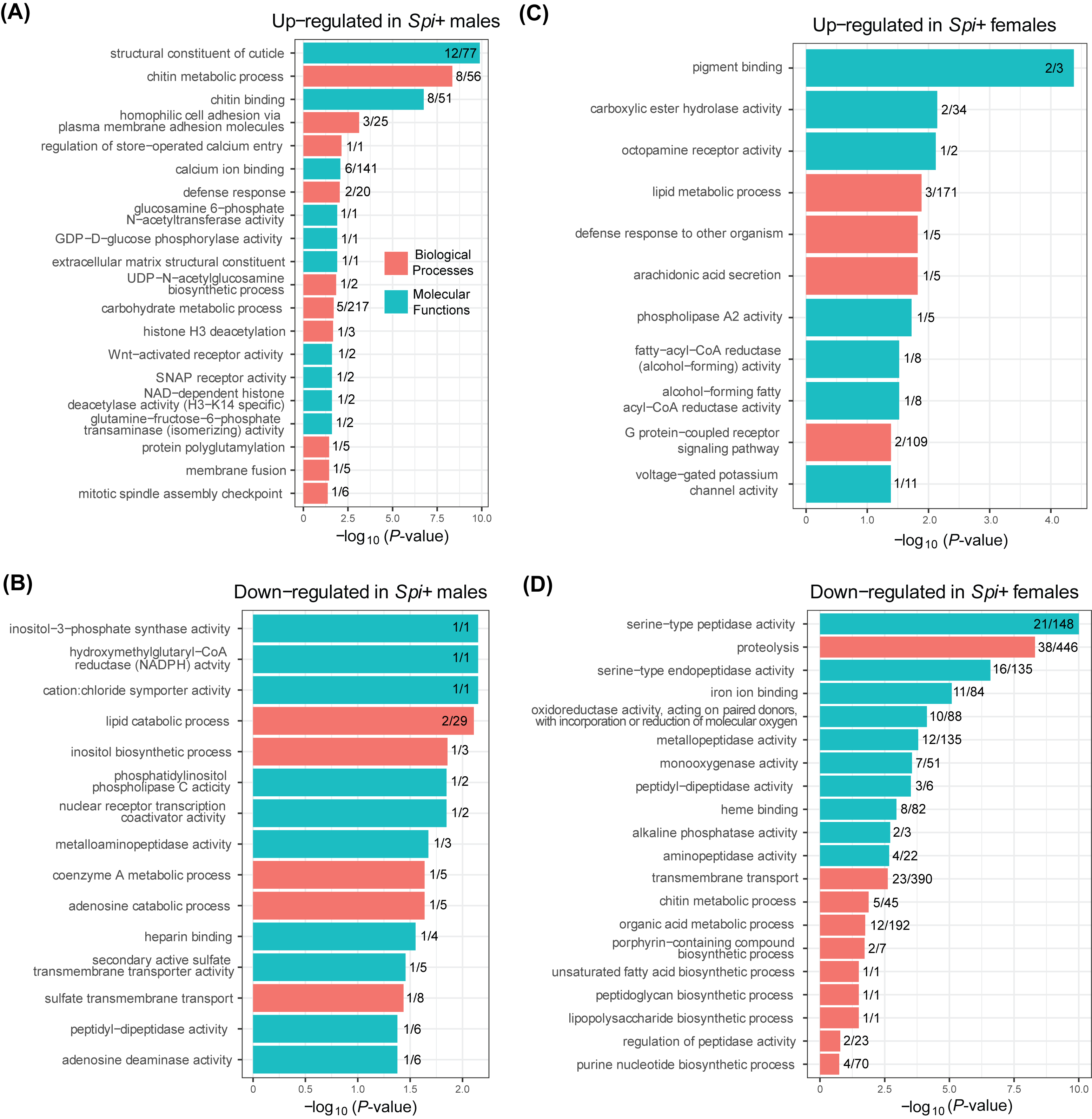

Supplement: S1 Fig — The bar diagrams show the significantly enriched GO terms among (A) the up-regulated and (B) the down-regulated genes in GffSpi+ males, and among (C) the up-regulated and (D) the down-regulated genes in GffSpi+ females. The number of genes associated with the corresponding GO terms to the number of genes belonging to that GO term within the entire set of genes in the genome is shown for each bar. The colors associated with the different bars denote the two different GO categories; BP: Biological Process, MF: Molecular Function. (TIF) [file ppat.1009539.s002.tif]

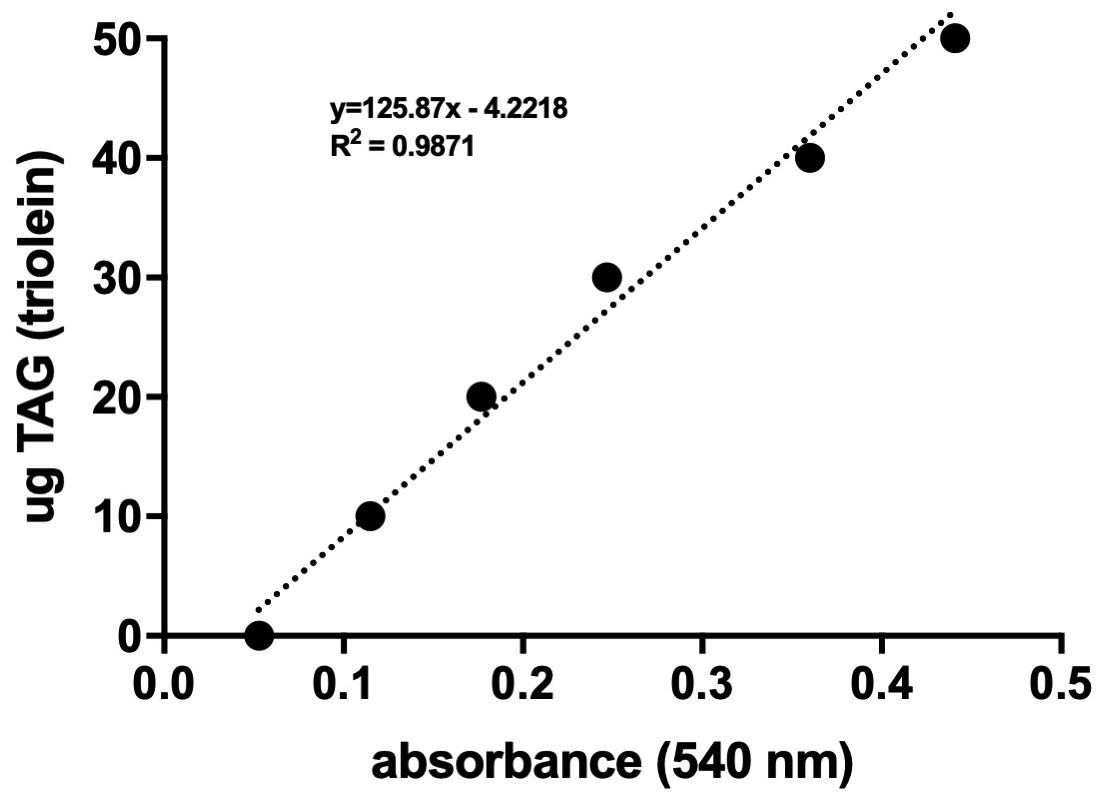

Supplement: S2 Fig — Pgrp-la expression in each sample was normalized relative to geometrical mean of tsetse’s constitutively expressed gapdh and β-tubulin genes. Each dot represents one biological replicate, and bars indicate median values. Statistical significance was determined via students t-test. (TIF) [file ppat.1009539.s003.tif]

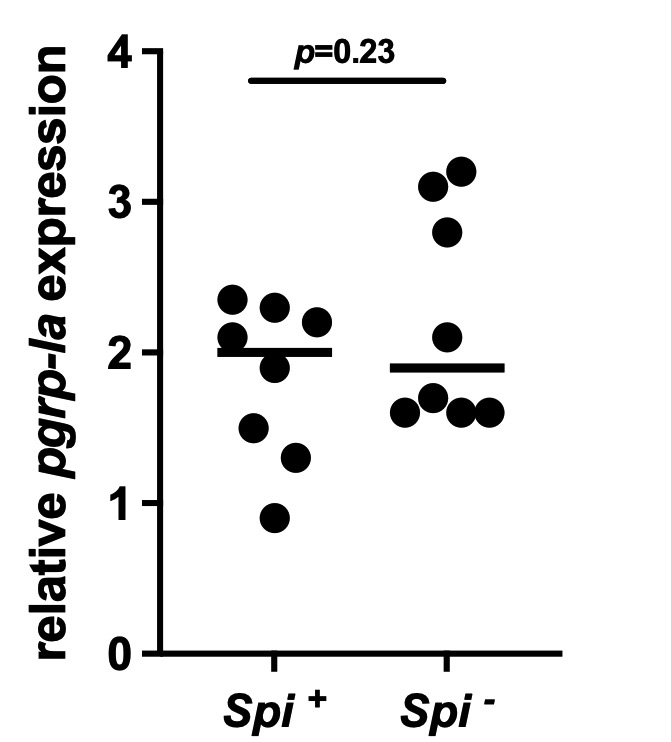

Supplement: S3 Fig — 0–50 μg aliquots of triolein were mixed with 100 μl of Infinity Triglycerides Reagent (Thermo Scientific) and samples were incubated at 37C for 10 min. Absorbance was measured at 540nm using a BioTek Synergy HT plate reader. (TIF) [file ppat.1009539.s004.tif]

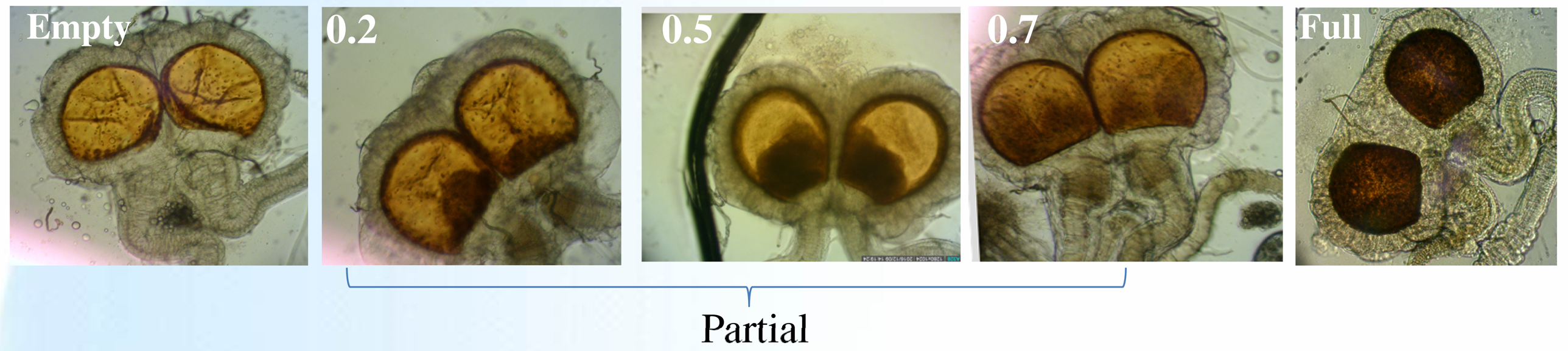

Supplement: S4 Fig — Image generated by Dr. Güler Demirbas-Uzel. (TIF) [file ppat.1009539.s005.tif]
