# Supplementary material for: Infection with endosymbiotic Spiroplasma disrupts tsetse (Glossina fuscipes fuscipes) metabolic and reproductive homeostasis
Source: PLoS Pathog. 2021 Sep 16;17(9):e1009539. doi: 10.1371/journal.ppat.1009539 (PMC8478229; doi:10.1371/journal.ppat.1009539)
Supplement: Attachment — Submitted filename: Son et al_Response to Reviewers.docx [file ppat.1009539.s012.docx]

25 August, 2021

Dear Dr. Lemaitre and PLoS Pathogens Reviewers,

We would like to thank you for your comprehensive review of our manuscript, entitled ‘Infection with endosymbiotic *Spiroplasma* disrupts tsetse (*Glossina fuscipes fuscipes*) metabolic and reproductive homeostasis’. In the following pages we provide an italicized response to each of the reviewer comments, which are listed in the order they originally appeared. We also provide two versions of the revised manuscript, one with tracked changes and one ‘clean’ version.

Again, we thank the editor and reviewers for taking the time to read the manuscript and provide valuable feedback about our research, and we hope the manuscript is now suitable for publication in PLoS Pathogens.

Sincerely,

Jae Hak Son, Brian Weiss, Daniela Schneider, Kisweda-sida Dera, Fabian Gstöttenmayer, Robert Opiro, Richard Echodu, Norah Saarman, Geoffrey Attardo, Maria Onyango, Adly Abdalla, and Serap Aksoy

**Part I - Summary**
Please use this section to discuss strengths/weaknesses of study, novelty/significance, general execution and scholarship.

Reviewer #1: This manuscript first describes the effect of Spiroplasma infection on tsetse fly reproductive tract transcriptome. Authors then performed functional experiments to assess the impact of the infection on both male and female reproductive ability. They conclude about a negative impact of Spiroplasma infection on both female and male reproductive fitness.

This work is overall very insightful and makes a significant contribution to the field, and the manuscript itself is well written.

Reviewer #2: This manuscript analyses the interaction between Spiroplasma and tse tse flies. There are three components - RNAseq comparison of S+ and S- individuals, examination of some phenotype associations, notably life history and sperm mobility, and transmission patterns. All elements are certainly novel and given the importance of tse tse, timely and important.

The impact on sperm motility was interesting and a phenotype not seen before in symbiosis studies. The paternal transmission is also very interesting (though I have some technical caveats to clear up).

Potential weaknesses of the study:

-I found the link between the RNAseq observations and the experiments a little forced. The experiments aren't really testing strong predictions from the RNAseq in my mind. Thus, whilst I found all the components interesting, I wasn't convinced by the synthesis.

*Our RNAseq based observations indicate that Spiroplasma infection induces significant changes in the expression of specific sex-biased genes. However, we could only make broad assumptions about their impact on tsetse’s reproductive physiology based on their putative functions in other insects. As such, we chose to monitor fundamental reproductive parameters (i.e., lipid availability, gonotrophic cycle length, sperm abundance and motility). Now that we have a better idea about how Spiroplasma infection impacts tsetse reproductive physiology, we can go back and look at how individual differentially expressed, sex biased genes mediate these phenotypes.*

-the RNAseq material is from the field and is limited in sample size within each sex; this raises the potential for confounding influences between S+ and S- individuals, which is particularly important in this study, as the most interesting comparisons are S+ vs S- within sex.
*The reviewer is correct in stating that the limited size of our field samples could bring some confounding effects into the results. However, as we mentioned above, we used our RNA-seq data to provide broad insight into the impact of Spiroplasma infection on the reproductive physiology of Gff males and females. This data then informed us about what sex specific functional experiments to perform.*

-For the transmission (and other) studies- Spiroplasma infection status is ascertained from endpoint PCR. Previous work has shown quite high variability in Spiroplasma titre in tse tse- potentially making more sensitive qPCR important for diagnosis of infection. This is important in RNAseq experiments and critical in transmission experiments, where one might diagnose paternal transmission in cases where the mother in fact has a low titre infection. Within this, I'd also like to know if leg snips have been validated as a means of diagnosing Spiroplasma status - in particular is there a false negative rate.

*To confirm the accuracy of our endpoint PCR analysis for the vertical transmission experiments, we randomly selected 10 Spiroplasma negative samples and 2 Spiroplasma positive samples (as determined by endpoint PCR using genomic DNA extracted from legs of Gff mothers) and performed qPCR. This procedure is now described in the Materials and Methods (subsection Laboratory maintenance of Gff and determination of Spiroplasma infection status). Our results, shown in S5 Data of the revised version of the manuscript, confirm our endpoint PCR analysis by showing no amplification of Spiroplasma DNA in our negative samples.*

-Also in the transmission measures - L416 Technically, if you have paternal and maternal transmission, you can only work out maternal vertical transmission rates in cases where the female is infected and the male is known to be uninfected, and in most cases, looking at the supplementary material data, the male isn't directly tested in these experiments. Thus, the experiments may overscore maternal inheritance.

*The reviewer makes a valid point here, which is that we can’t state for sure that Spiroplasma was maternally transmitted if we don’t know the status of the fathers. We have made the following changes to the manuscript to address this issue:*

*1) We have used our leg snip PCR assay to determine the Spiroplasma infection status of fathers in experiment 1. We already had this data for what was experiment 3 in the original version of the manuscript.*

*2) We could not determine the Spiroplasma infection status of fathers from experiment 2 because we did not save those flies. Thus, data from experiment 2 (now experiment 3 in the revised manuscript) has been made supplemental (see more about this below), and what was previously experiment 3 is now experiment 2. Maternal and paternal transmission data from experiments 1 and 2 is now combined and presented in one table (Table 1).*

*3) Our experiment 3 (previously experiment 2) data does not include the Spiroplasma infection status of fathers. Thus, to avoid making inaccurate claims, we describe this data more generally in the context of overall vertical transmission efficiency. To do so we state in the Results that this experiment confirms the steadfast nature of Spiroplasma maternal transmission, and may further confirm the occurrence of paternal transmission (because we see Spi+ offspring from Spi- mothers).*

*4) We have rewritten the corresponding Results subsection ‘Vertical transmission of Spiroplasma’ to reflect our new data and style of data presentation.*

Reviewer #3: This paper is centered on Glossina fuscipes fuscipes a tsetse species, which is a prominent vector of human African trypanosomiasis. The aim is to analyze and characterize the effects of the Spiroplasma endosymbiont on the reproductive biology at the physiological and metabolic levels. This aim is strongly based on previously acquired knowledge: 1) Spiroplasma, in Drosophila females affects fecundity as a a consequence of nutritional competition between the fly and bacterium for metabolically important lipids. 2) Populations of G. fuscipes are polymorphic for the presence of this symbiont. 3) Spiroplasma enhances refractoriness to trypanosomes, but the mechanism is unknown. It follows that the knowledge acquired from this study will be of extreme interest both for understanding the biological role of the presence of Spiroplasma in G. fuscipes and eventually for exploiting this symbiont for applicative aspects related to the control of this vector species. Indeed, the authors find that Spiroplasma competes for finite nutrients negatively impacting female fecundity. Moreover in males Spiroplasma affects the motility of sperm impacting their transfer to female spermatheca.
These data validate the authors hypothesis and support the extreme importance and novelty of this work.
One point of weakness of this work is the way in which the authors describe the results (Results section). The data are presented in an overly discursive manner. This is a pity , as often this results in the authors and subsequently the reader losing the thread of the argument. The results should be presented in more efficient and succinct manner. Moreover, they include considerations that should be in the Discussion. This will avoid the repetitions that are present in Results and Discussion sections.
If this weakness is resolved, the paper is very important and should be considered for publication.

*We identified one long paragraph in the Results that contains content more appropriate for the Discussion. As such we deleted this from the Results and incorporated it into the Discussion. Additionally, we removed material from the Results subsection ‘Vertical transmission of Spiroplasma’ because it detailed methods already presented in the Materials and Methods section.*

**Part II – Major Issues: Key Experiments Required for Acceptance**
Please use this section to detail the key new experiments or modifications of existing experiments that should be absolutely required to validate study conclusions.

Generally, there should be no more than 3 such required experiments or major modifications for a "Major Revision" recommendation. If more than 3 experiments are necessary to validate the study conclusions, then you are encouraged to recommend "Reject".

Reviewer #1: My main concern is that authors draw their conclusion without accounting for other symbionts infecting the tsetse flies, which could have confounding effects. The obligate endosymbiont Wigglesworthia seems especially important in this case because of the nutritional complementation it provides to the fly (hence its effect on the fly reproductive fitness). I suggest authors to control for Wigglesworthia titer in their lab reared flies to determine whether the observed effects on fitness (i.e. increased length of the GC in females and decreased sperm motility in males) are due to a direct effect of Spiroplasma (if Wigg. titer remains unchanged upon Spiroplasma infection) or an indirect effect on the primary symbiont (if Wigg. titer decreases upon Spiroplasma infection).

*This suggestion by the reviewer to investigate whether Spiroplasma infection alters the density of tsetse’s other symbionts, which may account for the reproductive phenotypes we observed, is a very good one. To address this question we collected midguts (including the Wigglesworthia harboring bacteriome) from Gff^Spi+^ and Gff^Spi-^ females, extracted genomic DNA, and used RT-qPCR to quantify the relative abundance of Wigglesworthia and Sodalis specific genes between samples. We observed no significant difference in the relative density of these two endosymbionts in Gff^Spi+^ compared to Gff^Spi-^ females. This data is now described in the Results (subsection, Impact of Spiroplasma infection on female metabolism and reproductive fitness****)***

*and presented in Fig. 3C.*

Reviewer #2: I'd like to see qPCR performed to confirm the endpoint PCR underpinning the transmission experiments, and also the RNAseq (though here it may be better instead to check for Spiroplasma reads in the RNAseq reads).

*To confirm the accuracy of our endpoint PCR analysis for the vertical transmission experiments, we randomly selected 10 Spiroplasma negative samples and 2 Spiroplasma positive samples (as determined by endpoint PCR using genomic DNA extracted from legs of Gff mothers) and performed RT-qPCR. Our results, shown in S5 Data of the revised version of the manuscript, confirm our endpoint PCR analysis by showing no amplification of Spiroplasma DNA in our negative samples.*

*Additionally, we mapped our RNAseq reads to Spiroplasma’s genome to confirm the Spiroplasma infection status of the samples we used for this analysis. We observed in our male data sets that Spiroplasma reads accounted for an average of 0.003% of the mapped reads in our Spiroplasma negative samples and 0.04% of the mapped reads in our Spiroplasma positive samples. For the female data sets we found that Spiroplasma reads accounted for an average of 0.002% of the mapped reads in our Spiroplasma negative samples and 0.03% of the mapped reads in our Spiroplasma positive samples (all of this data is now presented in S5 Table in the S1 Appendix). Although we did find Spiroplasma reads in the negative samples they represent a significantly lower proportion of the total reads than do the Spiroplasma reads from the positive samples. We speculate that reads derived from other bacteria present in our Gff samples may have conserved regions that mapped to the Spiroplasma genome. Additionally, unpublished evidence suggests that portions of Spiroplasma’s genome may have incorporated in to the Gff chromosome (similar to what has occurred with Wolbachia in other tsetse species), which could also account for the results we present here. Thus, we still feel that our Spiroplasma negative samples do not house this bacterium (as also confirmed by RT-qPCR).*

I don't think it is feasible really for a revision, but would like to see a more controlled RNAseq using the lab colony to compare against the wild results - or some validation in lab colonies with qRT PCR. it would provide evidence of repeatable patterns and lack of confound.

*We agree with the reviewer that this experiment would be very useful for validating our field-based study and we plan to do this in the near future*.

Reviewer #3: The experimental planning is clear although it is too descriptive. The authors must justify why for some experiments they use a very reduced number of replicates such as, for instance, the case of the sperm motility test and other tests.

*As the reviewer requested, we have added more replicates to some of our experiments, including those that describe results of our sperm motility and sperm quantitation assays. We feel that the remainder of our experiments include sufficient replicates to perform robust statistical analyses.*

**Part III – Minor Issues: Editorial and Data Presentation Modifications**
Please use this section for editorial suggestions as well as relatively minor modifications of existing data that would enhance clarity.

Reviewer #1: Line 282 : accessary -> accessory.

*We have made this change.*

Line 427 : the second occurence of GC2 on this line should be GC1.
*We have made this change.*

Line 515 : the reference ([47]) seems misplaced.

*We have moved the reference to its correct position midway through the next sentence.*

Line 682 : my understanding is that pgrp-la has been used in this work as a housekeeping gene because has been used previously in the tsetse fly. In Drosophila however, this gene is immune-inducible, and the authors detected a mild Toll pathway activation the transcriptomics data upon Spiroplasma infection, which makes me wonder whether pgrp-la is a suitable housekeeping gene in this particular case. Can authors demonstrate that its expression is unchanged upon Spiroplasma infection ? If not, I recommend using an alternative gene for normalisation.

*We have quantified the expression of pgrp-la in all of the Spi^+^ and Spi^-^ samples we used for our qPCR experiments. Our results indicate that no significant difference (p=0.23) in the expression of this gene exists between samples derived from Spi^+^ and Spi^-^ samples. This data is now shown in S3 Fig. in the revised version of the manuscript.*

Fig 1B : I find this figure a bit difficult to grasp because sample details are provided only on the horizontal lines. Adding the symmetrical annotation at the bottom of the columns could help readers to assess the clustering more easily.

*We have added the symmetrical annotation to the bottom of columns.*

Fig 3A : I find the figure display a bit confusing and would prefer to see the individual points instead of boxplots, as it has been made for the two other panels.
Same comment goes for Fig 4C, where it is hard to see the individual points on the violin plots.

*For the data presented in figures 3A and 4C, many of the values within each data set are the same. As such, when plotted individually, the software (GraphPad Prism) puts the points immediately next to each other and they thus appear as a line instead of individual values. Below is an example of the left panel of Fig. 4C plotted as individual points. Accordingly, the box plots and violin plots in Figs 3A and 4C, respectively, are Prism’s default output style for presenting this type of data, and we have published data similar to that in Fig 3A the same way before (see reference 38). We feel that the current presentation style is the most effective way of displaying the data.*

Fig 3B : please indicate which correction has been applied to the multiple testing.

*We now indicate in the figure legend the multiple t-tests were corrected using the Holm-Sidak method.*

Supplementary data 1 and 2 : only tsetse-specific gene codes are indicated, which makes it very difficult for people from other fields to make use of these data. It would be helpful to add more indication on each gene, at least a column with their full name (or the name of their closest known homolog).

*We have added a new column to Supplementary data files 1 and 2 that provides the Gff gene annotation for each gene ID.*

Reviewer #2: L97-99 This Spiroplasma does not have an official name, I think, so avoid raising one here? Microbiological nomenclature has rules for naming stuff. Also, it isn't really a close relative of S. poulsonii - makes it sound monophyletic with it - it is in the next clade along really. If one looks at a phylogeny, the first thing you say is not that it is a S. poulsonii relative.

*The reviewer is correct in that this Spiroplasma has not been formally assigned a scientific name, and as such, we have changed the manuscript so that the bacterium is referred to only as Spiroplasma (from tsetse flies). We have also removed the statement that Spiroplasma from Gff is a close relative of S. poulsonii.*

L266 comprised not compromised.

*We have made this change.*

L406 and elsewhere: can we avoid using moms and dads? Mothers and Fathers, or Male and female parents.

*We have made these changes.*

L416 and elsewhere: Table legends need improving - more detail to allow the table to be read standalone please.

*We have provided more detail in the table legend.*

L466-468: bit of a logical contrast - sex dimorphism and expression in reproductive tissues in sex biased gene expression are sort of opposites. Dimorphism is a body wide thing where the same tissues express differently, reproductive tissues are different tissues in the two sexes behaving differently. Sex biased gene expression between reproductive tissues is inevitable, but not necessarily linked to overall sexual dimorphism. Overall sex dimorphism and sex biased gene expression depends on the differences in male and female somatic phenotype - which varies between species.

*We appreciate the reviewer making this clarification and we have changed the manuscript wording accordingly. Specifically, we have changed ‘sexual dimorphisms’ to ‘sexual phenotypes’ (lines 467), and ‘sexual dimorphic phenotypes’ to ‘sexual phenotypes’ (line 174).*

L527: this logic is actually not true if there is paternal inheritance; if a symbiont has biparental inheritance it can invade the population without benefit (see Drosophila sigma virus as an example).

*In this sentence we have changed the word ‘must’ to ‘usually’.*

L537: did immunity fall out of the RNAseq analysis?

*The manuscript includes a subsection in the Results entitled ‘Immunity genes up-regulated in GffSpi+ males’, which provides an overview of the impact infection had on the male gonad (the bacterium had no impact on immune gene expression in the female gonad).*

L548-551: this argument makes absolutely no logical sense to me, sorry. Higher VT maintains symbionts more efficiently than low VT.

*We respectfully disagree with the reviewer in a situation such as this where the symbiont presents a parasitic phenotype that causes a significant reduction in its host’s fitness. Here we were trying to emphasize the unsustainable nature of a scenario where all flies in a population housed the bacterium (if steadfast vertical transmission of Spiroplasma did occur), which would eventually result in the elimination of both organisms as the result of reduced tsetse fitness. To make our point clearer, we have changed the sentences in question to read as follows:*

*The absence of steadfast vertical transmission of Spiroplasma by Gff could facilitate survival by reducing the population level detrimental impact (i.e., prolonged GC length and reduced sperm motility) associated with housing the bacterium. This may represent another advantageous factor that facilitates Spiroplasma’s ability to sustain infections within Gff populations across multiple generations.*

L644 16S not 16s- S is a unit - the Svedberg - and is capitalised.

*We have made this change.*

Reviewer #3: A major issue is the way in which the results are described. They must be much more concise in order that the data appear clear in their logical sequence and biological meaning.

*We identified one long paragraph in the Results that contains content more appropriate for the Discussion. As such we deleted this from the Results and incorporated it into the Discussion. Additionally, we removed material from the Results subsection ‘Vertical transmission of Spiroplasma’ because it detailed methods already presented in the Materials and Methods section.*
